# A Spectrophotometric Study of Plumage Color in the Eared Dove (*Zenaida auriculata*), the Most Abundant South American Columbiforme

**DOI:** 10.1371/journal.pone.0155501

**Published:** 2016-05-23

**Authors:** Diego Javier Valdez, Santiago Miguel Benitez-Vieyra

**Affiliations:** 1 Instituto de Diversidad y Ecología Animal (IDEA-CONICET-UNC), Centro de Zoología Aplicada, Universidad Nacional de Córdoba (UNC), Córdoba, Argentina; 2 Instituto Multidisciplinario de Biología Vegetal (IMBIV-CONICET), Facultad de Ciencias Exactas Físicas y Naturales, Universidad Nacional de Córdoba (UNC), Córdoba, Argentina; Hungarian Academy of Sciences, HUNGARY

## Abstract

For birds, plumage color perception is critical in social interactions such as courtship, in both monochromatic and dichromatic species. In the Eared Dove (*Zenaida auriculata*), perhaps the most abundant South American Columbiforme, the plumage of males and females looks alike and both sexes share the same melanistic coloration with gray and pink tones. The aim of this study was therefore to determine whether evident sexual dichromatism exists in the plumage of the Eared Dove using a spectrophotometry technique in the avian-visible range (300–700 nm). The results of the classic colorimetric variables analysis (hue, chroma and brightness) show that males are in general brighter and have higher UV chroma values than females. The avian visual model points to differences in achromatic and chromatic levels between males and females in body regions possibly involved in sexual selection (e.g. the crown). The model also indicates chromatic or achromatic differences in body regions not subject to sexual selection such as the black spots on the wing coverts and white tail bands, both of which may be involved in intra- or inter-gender-specific communication.

## Introduction

Plumage color is of critical importance to birds since it is linked to intra- and inter-specific communication processes [1–7]. At the intra-specific level, coloration has been studied in relation to communication between the sexes (sexual selection), the female usually choosing males with the brightest or most colorful plumage [3, 5, 8–12]. Furthermore, there is a close connection between the quality of a bird’s coloring and its physiological status, color being taken as a sign of fitness [5, 13–15]. The question then arises as to how individuals of different sexes are recognized in those species which do not have a distinctive sexual dichromatism and in which the coloration of male and female plumage is apparent to the human eye, as in the case of the Eared Dove (*Zenaida auriculata*). This Columbiforme sub-species of medium size (20-24cm long) mainly inhabits the southern region of South America [16] and for over three decades has been considered a plague [17–19]. It has a melanistic coloration type with slightly pink and gray tones, black spots on its face and wings, golden iridescent feathers on the side of the neck, and a gray tail with a black sub-terminal band and white outer tip [20]. So far in this species, there are not works that report an objective analysis of plumage color, since the crown of the adult male appears to be slightly lighter blue and the belly pinker than the female. Like most diurnal bird species studied, Columbiformes are in general well-adapted to color vision, possessing four types of cone photoreceptor cells in their retinas (most mammals including humans have only three) [21–24] which pprovide them with a different perception of plumage color from humans [25]; specifically, Columbiformes has a short-wave cone that allows them to see the UV portion of light spectrum [26, 27]. Numerous studies have shown that both the UV component and overall color are important in social interactions of birds, especially in male-female interactions during the breeding season (sexual selection), since females prefer males that are brighter and more colorful [3, 5, 8, 9, 11].

Although the reproductive ecology of the Eared Dove is known in detail [28, 29] and some aspects of courtship suggest that visual signals play an active role in female choice, there are no objectives studies reporting intersex differences in plumage color for this species. The aim of the present study was therefore to determine whether or not adult individuals of the Eared Dove (*Z*. *auriculata*) manifest an evident dichromatism. To this end we used the spectrophotometry technique to objectively measure the plumage reflectance of 12 body regions across the visible spectrum of birds (300–700 nm).

## Materials and Methods

This study was carried out in strict accordance with the Guidelines for Ethical Research on Laboratory and Farm Animals and Wildlife Species and with the prior approval of the ethics committee of the Consejo Nacional de Investigaciones Científicas y Técnicas (CONICET) (Resolution No. 1047 ANNEX II, 2005). The necessary permits were acquired from the Ministry of Environment of the Province of Córdoba, Argentina, through the General Directorate of Natural Resources, to capture specimens of Eared Dove for scientific purposes.

### Animals

Following the procedure described in Navarro et al. 1992 [30] 20 adult male, 12 adult female and 4 juvenile specimens of Eared Dove (*Z*. *auriculata chrysauchenia*) were captured in the grounds of the Córdoba Zoo, Argentina (31°25’31.79"S, 64°10’29.92"W) during the months of November and December 2014, thus avoiding the molting period (April to August). The feathers of freshly killed individuals were used in the experiments. Animals were sacrificed with an intra-peritoneal injection of concentrated chloral hydrate solution and sexed by examination of the reproductive organs following the procedure described in Bucher et al. 1980 [28]. The plumage color of 12 body regions was analyzed: crown, nape, iridescent neck feathers, back, chest, belly, wing coverts, primary remiges, secondary remiges, black spot, black tail band, white tail band.

### Spectrophotometric measurements and plumage color

Reflectance in the avian spectral range (300–700 nm) was measured using an Ocean Optics USB4000 spectrophotometer equipped with a halogen and deuterium light source (830 Douglas Ave., Dunedin, FL, USA 34698), both connected to the sensor by a bifurcated fiberoptic cable. The plumage was illuminated and reflected light collected at 45° to the surface of the feather in order to avoid specular reflectance [31]. The probe was mounted in a prismatic probe holder held over the selected region of the study skin. The distance between the probe and the plumage was 4 mm, the spectrophotometer resolution 0.19 nm, the integration time 300 msec and each spectrum was the average of three readings. A white standard (Ocean Optics, WS-1-SS White Standard) was used to re-calibrate the equipment between each measurement in order to correct for possible shifts in performance. Reflectance was measured using SpectraSuite software (Ocean Optics,Inc.). Measurements were carried out blind to the sex.

Two complementary approaches were used to determine plumage color in the Eared Dove using the *pavo* package [32] of R software (R Core Team 2015 [33]): classic colorimetric variables analysis (hue, chroma and brightness) and an avian visual model. For the latter, cone quantum catch (Q) for each of the four avian cones was calculated under a standardized daylight illumination (D65) as a representative spectrum for open habitat midday ambient light. Although cone parameters have not been measured in *Z*. *auriculata*, the generalized spectral cone sensitivities of VS-type avian eyes was used since this visual system characterizes all Columbiformes studied so far [34]. The sum of the two longest-wavelength cones was used to calculate achromatic cone stimulation.

The relative cone excitation values were then used to calculate the coordinates of body parts in a tetrachromatic color space [35]. Finally, in order to estimate the chromatic and achromatic contrasts among different body regions a model of avian vision was applied which assumes that receptor noise limits discrimination in each cone [36–38]. Contrasts were characterized in units of "just noticeable differences" (JND), such that one JND represents the threshold of possible discrimination (See S1 Text and S1 Table for more information).

As only four juveniles were captured, none of which differed from females in terms of spectrophotometric characteristics, it was decided to restrict the study in juveniles to the classic colorimetric variables.

### Statistical analyses

The reflectance spectra from males, females and juveniles were examined to determine the presence or not of overlapping regions. Further analyses were carried out in plumage regions where spectra did not completely overlap.

One-way ANOVA was used to test for significant differences in classic colorimetric variables (hue, chroma and brightness). Differences among coordinates in the tetrahedral color space were examined using MANOVAs and post-hoc Hotelling's tests.

Finally, to address whether distances between sexes were greater than would be expected by chance, the mean (± SE) of all pair-wise chromatic and achromatic distances (measured in JNDs) among males, among females and between males and females, was calculated for every body part. A multi-response permutation procedure (MRPP) was then applied where the observed pair-wise mean distance between sexes was compared with a distribution obtained by randomly assigning a sex to the individuals. One thousand pseudo-values were obtained in this way, and the observed value was considered significant if it was greater than 95% of the pseudo-values.

## Results

### Reflectance spectra and classic colorimetric variables

**Fig 1:**
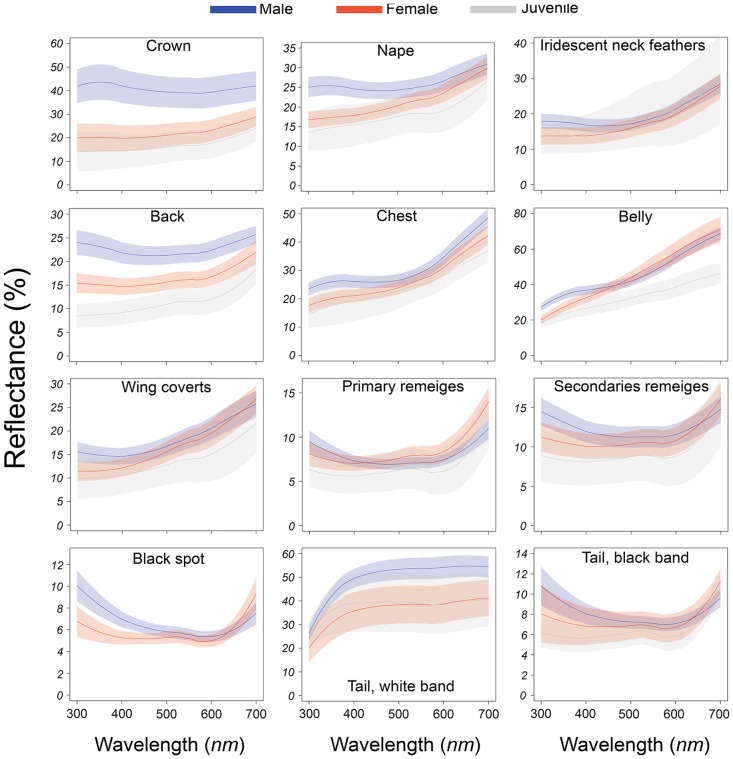
Reflectance spectra of the twelve Eared Dove body regions. Each spectrum represents mean reflectance ± 2SE of 20 males in blue, 12 females in red and 4 juveniles in gray.

The reflectance spectra for the twelve body regions studied fell in the range of 300 to 700 nm (Fig 1).

In seven regions (crown, neck, back, chest, belly, black spot on the wing coverts and white tail band) bands of ±2 standard errors did not overlap in at least part of the reflectance spectra. A particularly marked contrast was found between male and female spectra in three regions: crown, back and white tail band. The upper right panel shows reflectance spectra of iridescent neck feathers from males, females and juveniles of the Eared Dove. It was decided to discard iridescent feathers from the analysis since the characteristic multiple reflectance peaks were not observed, possibly due to the fact that the spectrophotometer used in this study, with a bifurcated fiber-optic probe to illuminate and collect the reflected light and the angle of incidence of light were not appropriate for these feathers.

Table 1 shows the classic colorimetric variables studied for seven body regions where differences in reflectance spectra between males, females and juveniles can be observed.

**Table 1:** Classic colorimetric variables: hue, UV-Chroma and brightness.

	*Hue*				*UV-Chroma*				*Brightness*			
	♂	♀	J	*F* and *p*	♂	♀	J	*F* and *p*	♂	♀	J	*F* and *p*
***Crown***	465±40^a^	**↑**700^b^	**↑**700^b^	*F*_(2.33)>_12.57*p*<0.0001	0.26±0.004^a^	0.22±0.007^b^	0.21±0.017^b^	*F*_(2.33)>_17.51*p*<0.0001	40.82±3.35^a^	22.04±2,51^b^	16.65±3.42^b^	*F*_(2.33)>_10.69*p*<0.0001
***Nape***	**↑**700^a^	**↑**700^a^	**↑**700^a^	*F*_(2.34)_ = 1.29*p* = 0.28	0.245±0.006^a^	0.205±0.007^b^	0.203±0.007^b^	*F*_(2.34)_>11.42*p*<0.0001	25.88±1.13^a^	21.19±1^b^	17.94±2.57^b^	*F*_(2.34)>_7.09*p*<0.0027
***Back***	539±45^a^	633±45^a^	**↑**700^a^	*F*_(2.33)_ = 1.67*p* = 0.2	0.26±0.005^a^	0.23±0.008^b^	0.196±0.01^b^	*F*_(2.33)_>9.03*p*<0.0001	22.55±0.1^a^	16.34±0.86^b^	11.21±1.33^b^	*F*_(2.33)_> 16.98*p*<0.0001
***Chest***	**↑**700^a^	**↑**700^a^	**↑**700^a^	*F*_(2.34)_ = 0.38*p* = 0.68	0.209±0.003^a^	0.187±0.005^b^	0.177±0.011^b^	*F*_(2.34)_>9.49*p*<0.0001	30.69±1.18^a^	26.43±1.23^a^	23.96±2.87^b^	*F*_(2.34)_>4.56*p*<0.017
***Belly***	**↑**700^a^	**↑**700^a^	**↑**700^a^	*F*_(2.34)_>0.38*p*<0.68	0.183±0.003^a^	0.15±0.006^b^	-	*F*_(2.34)_>18.01*p*<0.0001	45.64±1.36^a^	45.28±1.87^a^	32.64±2.33^b^	*F*_(2.34)_>7.89*p*<0.0016
***Black spot***	-	-	-	-	0.317±0.012^a^	0.259±0.017^b^	-	*F*_(2.30)_>7.84*p*<0.008	6.59±0.28^a^	5.70±0.27^b^	-	*F*_(2.30)_>4.37*p*<0.044
***Tail*, *w*.*b*.**	-	-	-	-	-	-	-	-	50.21±2.01^a^	36.44±3.82^b^	38.18±5.76^b^	*F*_(2.33)_>4.37*p*<0.044

Differences among groups were tested with ANOVAs. Different letters indicate significant differences among bird groups (males, females, and juveniles), Tukey test, p<0.05. ↑ = above 700 nm. Data are Mean ± SE. J means “Juveniles”. Different letters (^a^ and ^b^) indicate significant differences between groups.

The body region with most intersex differences was the crown, with a hue of 465±40 nm for males (blue chroma: ♂ 0.27±0.001, 0.25±0.003 ♀ and juveniles; red chroma: ♂ 0.24±0.004, ♀ 0.29± 0.008, juveniles 0.3±0.018) and above 700 nm for females and juveniles). Hue values for the back were 539±45 nm for males, 633±45 nm for females and above 700 nm for juveniles, indicating that males are more light blue and females and juveniles brown (see also Fig 1). The rest of the body regions studied showed no differences in hue and were brown-gray, black or white (see Fig 1, Table 1). UV chroma values were in all cases higher in males than in females and juveniles (see Table 1). Males were observed to be brighter than females and juveniles, except in the chest and belly, where juveniles were brighter.

### Avian visual model and comparisons between sexes

**Fig 2:**
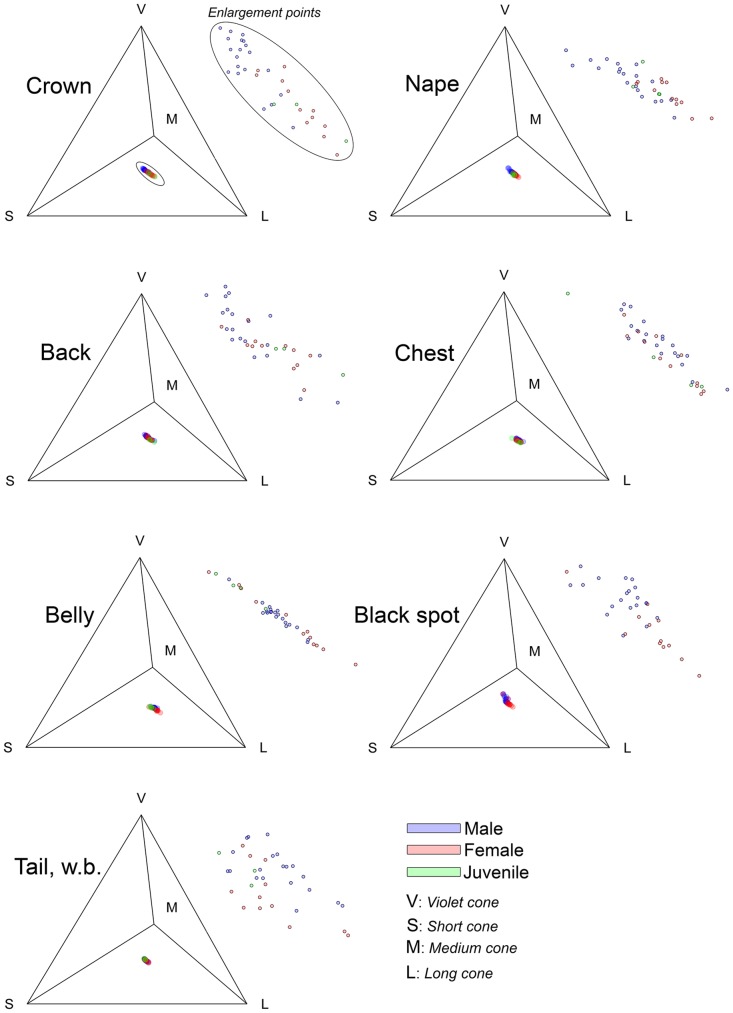
Tetrahedral colored space for seven Eared Dove body regions where significant differences between males, females and juveniles were observed. Enlargement points are shown to the left of each tetrahedran.

Inspection of the relative cone absorbance of the light reflected by the seven body regions in Goldsmith's tetrahedral color space revealed significant differences between males and females (Fig 2).

In general it can be observed that females reflect more of the energy reaching the red cone than males (MANOVA, in all cases Wilk's λ> 0.57, approximate *F>* 3.2, df = 6; 60, *p<* 0.0085).

Despite the differences found in the coordinates x, y, z of each individual in the tetrahedron color space, once a model of avian vision was applied, the crown was the only body region which exhibited significant differences between the sexes in both chromatic and achromatic distance (JNDs). Neck, chest, belly and black spot revealed significant differences between males and females only in chromatic distance whereas black tail band and white tail band only showed significant difference in achromatic distances (Table 2).

**Table 2:** Color distance in just noticeable differences (JNDs).

	*Chromatic Distance* (JNDs)			*Achromatic Distance* (JNDs)		
	♂♂	♂♀	♀♀	♂♂	♂♀	♀♀
***Crown***	(0.91±0.05)	2.21±0.07*	1.30±0.1	5.64±0.3	8.33±0.35*	5.15±0.48
***Nape***	1.23±0.06	1.68±0.07*	(0.95±0.07)	3.68±0.2	3.5±0.16	2.86±0.25
***Back***	1.19±0.06	1.39±0.05	1.15±0.09	3.28±0.18	4.67±0.19*	3.20±0.26
***Chest***	(0.83±0.03)	1.1±0.03*	(0.92±0.06)	2.87±0.14	2.86±0.14	2.69±0.26
***Belly***	(0.67±0.03)	1.61±0.05*	1.63±	2.26±0.12	2.52±0.13	2.82±0.28
***Black sopt***	1.51±0.07	2.24±0.08*	1.75±0.18	3.35±0.17	3.2±0.15	3.27±0.29
***Tail*,*w*.*b*.**	(0.69±0.03)	(0.71±0.03)	(0.76±0.07)	3.13±0.16	6.13±0.23*	4.95±0.46

♂♂ = comparison between males; ♂♀ = comparison between males and females; ♀♀ = comparison between females. Values below 1 are not discriminatory and are shown in parentheses (e.g. 0.91 = individuals cannot be distinguished one another.). A Multi-Response Permutation Procedure was used to test whether or not color the distance between males and females was greater than would be expected by chance. Significant differences (P<0.05) are indicated with *.

## Discussion

This paper reports the first objective and quantitative data pointing to the existence of manifest dichromatism between males and females of the Eared Dove (*Z*. *auriculata*), perhaps the most abundant member of the *Zenaida* genus in South America [39].

The classic colorimetric variables analysis (hue, chroma and brightness) revealed that in the seven body regions where significant differences between genders were observed, the plumage of the males was in general brighter than that of the females and juveniles; males also showed higher UV chroma values than females and juveniles and for the crown and back only differences in tone between males, females and juveniles (males more grayish blue, females and juveniles grayish brown) were observed. This finding is of interest since most of these regions (crown, neck, back, chest) are the ones displayed during courtship: Eared Dove presents the typical bowing display common to most doves and pigeons [40], the male chasing the female with inflated neck, bowing motions and the typical coo vocalization. Our findings suggest that some of these body regions may be subject to sexual selection, a hypothesis further reinforced by the chromatic and achromatic distance values for the crown and back in the avian visual model. All males have a similar crown color (grayish blue) with differences in brightness, whereas in females both color and brightness of the crown can vary (slightly blue to brown, Fig 1 left top panel). Differences observed in the back are primarily in terms of brightness. These results indicate that it is primarily the males that are subject to selection pressure. Similar results were reported by Mahler and Kempenaers in another South American Columbiforme, the Picui Dove (*Columbina Picuí*); these authors demonstrated that the males´s neck and wing stripes are brighter than those of females [41]. Picui Dove males, like Eared Dove males, have higher UV reflectance values associated with courtship body regions, suggesting that these regions in both species would be subject to sexual selection for greater sexual dichromatism. Other interesting results presented here are those obtained for body regions such as the black spot of the wing coverts and white tail band. The analysis of classical colorimetric variables (UV-chroma and brightness) shows higher values of UV-chroma and brightness for males than for females and the chromatic and achromatic distances given by the avian visual model show significant differences at the chromatic level for the black spot of the wing coverts and differences in achromatic distances in tail white band between the sexes. Unlike other Columbiformes species such as Rock Dove (*Columba livia*), where the male can spread his tail feathers in a fan during the "bowing display" [40], the Eared Dove does not expose these structures during courtship, possibly indicating that these body regions are involved in intra-specific communication processes other than communication between the sexes, perhaps intra-gender competition.

Feather iridescence is a phenomenon that manifests itself in various bird groups [42–45]. The iridescent neck feathers displayed by the Rock Dove during courtship have multiple reflectance peaks at different wavelengths [43]. In our study, iridescent neck feathers of the Eared Dove showed no such reflectance peaks (for which reason they were discarded from the analysis; see results), although this area of plumage is displayed during courtship, perhaps as part of the sexual signal emitted by the males to attract females. This apparent lack of multiple reflectance peaks in the iridescent feathers of the Eared Dove could be due to technical limitations; there is evidence showing that the spectrophotometer approach used here, employing bifurcated fiber optics to illuminate and collect the reflected light, is not suitable for studying plumage iridescence [46]. Further studies are therefore required on this body region using a different optical fiber arrangement, angles of light incidence and an analysis of the area size in order to ascertain its participation in sexual display.

In conclusion, this study reports a manifest sexual dichromatism in what is perhaps the most abundant Columbiforme in South America, the Eared Dove (*Z*. *auriculata*), where males are brighter and show chromatic and achromatic differences with respect to females, suggesting a selection pressure towards males. We also demonstrate for the first time that the spectrophotometry of juvenile plumage is more like that of females, with a color tending towards cryptic. Though this study is of a descriptive nature, it opens the way for new hypotheses and interrogatives with a view to elucidating the relationship between vision, plumage color and successful reproductive capacity in this species.

### Avian visual model and intrinsically photosensitive retinal ganglion cells (ipRGCs)

The avian visual model is a valuable tool for interpreting color. New evidence has emerged in recent years indicating the involvement of a new cell group for the processing of visual information (color perception and image formation): intrinsically photosensitive retinal ganglion cells (ipRGCs) [47–49]. These cells express a photopigment called melanopsin which has its absorption peak between 480 and 490 nm in birds [50, 51]. The participation of these ipRGCs in image forming and color perception in a behavioral and ecological context has not been evaluated; the development of experiments to elucidate this issue will therefore be useful in incorporating new elements into the avian visual model.

## Supporting Information

S1 Text Valdez DJ routine work. Routine work in R (script) showing the different steps performed to analyze the data. For all body regions (except for the crown.) must be eliminated step four in the routine work.
(R) Click here for additional data file.

S1 Table Crowntable.txt. Table (example) containing wavelength and spectrophotometric values of the crown of males, females and juveniles. To download all the body regions data follow this link: https://dx.doi.org/10.6084/m9.figshare.3364681.
(TXT)
